# The effects of an acute bout of exercise on neural activity in alcohol and cocaine craving: study protocol for a randomised controlled trial

**DOI:** 10.1186/s13063-018-3062-0

**Published:** 2018-12-29

**Authors:** Flora Colledge, Sebastian Ludyga, Manuel Mücke, Uwe Pühse, Markus Gerber

**Affiliations:** Department of Sport, Exercise and Health, University of Basel, Birsstrasse 320b, 4052 Basel, Switzerland

**Keywords:** Addiction, Exercise, Inhibitory control, Neuroimaging

## Abstract

**Background:**

Numerous studies suggest that exercise may be an effective adjunct treatment for substance use disorders. It has been suggested that exercise-induced improvements in inhibitory control may reduce craving for the substance of abuse. However, this potential mechanism has seldom been researched.

**Objectives:**

The aim of the ExAlCo Study is to examine how acute bouts of exercise, at varying intensities, impact on craving for cocaine or alcohol. Cerebral haemodynamic responses during cognitive tests of inhibitory control, and exposure to substance-related cue imagery, will also be assessed using functional near-infrared spectroscopy.

**Design:**

The study is a crossover randomised controlled trial. Participants will be recruited from inpatient and outpatient psychiatric treatment centres, on the approval of their treating physician. A healthy control group will be recruited using online advertising. All participants will undergo each of three conditions in randomised order: 20 min of cycle ergometry at 50–60% of maximum heart rate; 20 min of exercise at 70–80% of maximum heart rate; and 20 min of quiet reading. Immediately before and after each condition, participants will be asked to complete a computerised Stroop test, watch a film containing substance-related images and self-report craving levels. During the Stroop test and film viewing, participants’ neural activity will be measured via functional near-infrared spectroscopy.

**Outcomes:**

The primary outcome measures are self-reported craving, inhibitory control and cerebral haemodynamic response to the Stroop test and a substance-related film. It is hoped that the findings from this study will shed more light on the role of exercise in the treatment of substance use disorders, particularly its scope in preventing relapse through reduced craving severity.

**Trial registration:**

ClinicalTrials.gov, NCT03502486. Registered retrospectively on 5 April 2018.

**Electronic supplementary material:**

The online version of this article (10.1186/s13063-018-3062-0) contains supplementary material, which is available to authorized users.

## Background

Craving substances to which an individual has developed dependence is one of the most troubling symptoms of addiction, and is frequently reported by sufferers to lead to relapse following a period of abstinence [1]. This holds true for cocaine-dependent [2] and alcohol-dependent [3] individuals. Craving for a substance is therefore a subject of great importance in addiction research and can be assessed in a number of ways, frequently via exposing the individual to the substance of dependence, or its image or scent, a so-called “cue” [4]. Alcohol-dependent and cocaine-dependent individuals reliably report enhanced craving and display physiological reactivity to their respective substance cues [5, 6].

Neuroimaging techniques are frequently used to examine the areas of the brain which are involved in the experience of craving [7, 8]. Because craving is an important outcome in addiction treatment, insight into functional brain activity serves to guide treatment innovations and improve understanding of the physiological substrates of addictive behaviour [9]. While a number of brain regions are implicated in cue reactivity and craving, there has been a growing focus on the prefrontal region of the outer cortex [7, 10]. Bonson et al. [11] assessed neural activity in response to cocaine cues in cocaine-dependent individuals, and determined that increased activation of the lateral orbitofrontal cortex and dorsolateral prefrontal cortex correlated with craving. Similarly, Grüsser et al. [12] reported activation of the medial prefrontal cortex in abstinent alcoholics following exposure to alcohol cues, with more intense activation observed in those who subsequently relapsed.

The prefrontal cortical region is also implicated in a number of cognitive processes, including inhibitory control [13]. Inhibitory control is a core component of executive function, and is defined as the ability to control one’s attention, behaviour and thoughts, and to override an internal predisposition or ignore external stimuli [14]. Crucially, inhibitory control has also been shown to be a relevant parameter in the understanding of craving, as it constitutes the ability to control a reaction upon exposure to the craved substance [15]. For example, Goldstein and Volkow [16] report that the activation of the orbitofrontal cortex observed during craving is associated with reward reinforcement, but also with deficits in inhibitory control. The ability to exercise inhibitory control during exposure to cocaine cues has been shown to rely in part on normal functioning of the prefrontal cortex [17]. Neural functioning in the prefrontal cortex therefore appears to be relevant to the phenomenon of craving, and inhibitory control may be a moderating factor [18].

A number of techniques can be used to produce images of prefrontal cortical activity. Functional magnetic resonance imaging (fMRI) enables imaging of deep brain structures, and allows for highly accurate spatial localisation of brain activity. However, the method has two chief limitations. First, fMRI requires the participant to lie, without moving, during the imaging process; consequently, it is unclear how apparent reductions in craving in this rather unnatural state will translate to craving experienced in a more normal setting. Second, fMRI must be carried out in a specialist laboratory, and the machine itself is large and extremely costly. Hence, it cannot be expected that findings from fMRI studies can be easily translated to the practice of non-hospital-based addiction treatment services [19]. By contrast, functional near-infrared spectroscopy (fNIRS) is a portable, wearable imaging device, which is much less susceptible to movement artefacts [20]. Both imaging systems identify neural activity by monitoring changes in the concentration of blood oxygen levels in various brain regions [21]. As fNIRS is mainly suited to assessing neural activity in the outer cortex of the brain, for studies focusing on cognitive functions it represents an affordable, portable means of assessment. As the device can be positioned on the head within 2 min, and does not require the application of conductive gel, it is also more time efficient than EEG. These properties of fNIRS mean that, if it proves a useful tool in the assessment of craving severity in addiction, it can be incorporated into clinical practice with comparative simplicity [22].

For the purposes of the present study, assessing neural activity during exposure to substance cues permits a physiological assessment of cue reactivity to accompany craving self-report, a measure which, on its own, may be subject to social desirability bias [23]. The fNIRS assessment during the Stroop test provides additional information on the processes underlying impaired or successful inhibitory control. Given that previous studies have linked exercise-induced benefits with prefrontal cortex function [24, 25], an examination of the cerebral haemodynamic response to the cognitive task may also allow deeper insights into the mechanisms by which exercise benefits inhibitory control in patients undergoing therapy for alcohol addiction and healthy adults.

fNIRS has already been used to assess brain activity in alcohol-dependent individuals. Ernst et al. [26] reported decreased concentration of deoxygenated haemoglobin (HHb) in the right dorsolateral prefrontal cortex during an approach-avoidance task, and increased concentration of oxygenated haemoglobin (HbO_2_) in the left anterior lateral orbitofrontal cortex in response to image cues. Schecklmann et al. [27] reported reduced activation in the frontotemporal region during a cognitive task. Importantly for the purposes of the present study, Bunce et al. [28] have used fNIRS imaging to show enhanced responses to alcohol cues in non-treatment-seeking alcohol-dependent patients (compared to social drinkers). In the sole study to examine craving for alcohol and cocaine, discussed in detail in the following, Grandjean da Costa et al. [29] found exercise-induced increases in oxygenated blood concentrations in the prefrontal cortex compared to baseline, accompanied by lower craving scores. Increases in oxygenation were, however, less pronounced than those in healthy controls.

Central to the focus of the present study, acute exercise has been shown to influence activity in the prefrontal cortex. In both men and women, cortical activity measured with electroencephalography has been found to increase across different regions from warm-up to moderate cycling exercise, but decreases in frontal regions with prolonged duration [30, 31]. Additionally, prefrontal cortex activity during exercise is affected by intensity as submaximal to maximal loads elicit a decrease in the oxygenation of the prefrontal cortex [32]. Due to the exercise-induced modulation of prefrontal cortex function, it can therefore be speculated that exercise may influence craving.

Exercise has increasingly been examined as a potential adjunct therapy in addiction treatment [33]. While most research has focused on the effects of multi-session exercise interventions, some studies have shown promising results of acute exercise bouts on addiction-related outcomes. In this respect, acute bouts of exercise have been shown to both reduce perceived craving and increase inhibitory control in methamphetamine-dependent individuals [18]. To date, evidence for exercise in alcohol dependence is weaker than for other substances [33, 34]. The reason for this discrepancy is not clear [35].

A potential underlying mechanism of exercise-induced reductions in craving might be an increase in inhibitory control after exercise. A recent meta-analysis of 40 studies has reported small to moderate effects of moderately intense aerobic exercise on inhibitory control and other components of executive functioning in healthy populations. The effects were independent of fitness levels, so that athletes and non-athletes can expect similar benefits [36]. Investigating further moderators with meta-regression, Chang et al. [37] have found that aerobic exercise bouts lasting 11–20 min elicit greater benefits for executive function than longer or shorter durations. Finally, Everitt [38] has identified an association between prefrontal cortical inhibitory control and extinction; that is, improved inhibitory control is associated with reduced cue-elicited craving. fNIRS studies with healthy adults were able to show that increased improved inhibitory control following aerobic exercise at mild to moderate intensity is associated with increased activation of the prefrontal cortex and the dorsolateral area in particular [25, 39]. In contrast, a decrease of the HbO_2_ concentration during highly intense aerobic exercise has been found to be correlated with slowing of reaction time on an inhibitory control task [40]. It therefore seems that fNIRS is an appropriate method for examining the potential effects of exercise on cue-elicited craving and inhibitory control.

To date, one study has examined the effect of an acute exercise bout on cocaine and alcohol craving, and has employed fNIRS during a test of inhibitory control to examine whether this may be a moderating factor. Grandjean da Costa et al. [29] reported reductions in cravings for alcohol or cocaine following a maximal exercise test; however, no significant improvements in reaction time on the Stroop test were observed. While this study is an important initial step in exploring whether, and to what extent, exercise-induced reductions in craving occur, and are moderated by inhibitory control, two aspects of the study design might obscure certain effects. First, an incremental maximal exercise test was used as the acute exercise bout; this means that all participants increased their exercise intensity from easy, through moderate to very intense, in a single unbroken sequence, and completed the craving assessment only before and after the entire bout. This means that it is impossible to tell what effect intense exercise has on craving, as the reported reductions may simply carry over from the moderate intensity preceded it. Second, the tests of inhibitory control were carried out repeatedly during the exercise test. While this enabled the researchers to obtain results at varying intensities, it is at least possible that the challenge of combining exercise (particularly at high intensities) with the task may have affected the results. It must also be noted that this study therefore suggests that exercising may reduce craving, and does not address the related but equally important question of whether craving following exercise is reduced.

In summary, there is evidence that craving in alcohol or cocaine-dependent individuals can be reliably elicited by exposure to images, and that craving correlates with specific activation patterns in regions of the prefrontal cortex. The activation of these regions can be imaged using fNIRS. Inhibitory control may moderate craving, and activation during inhibitory control tasks can also be imaged using fNIRS. Finally, during an acute bout of exercise, activity in the prefrontal cortex increases (at moderate intensities) and decreases (at high intensities), and acute exercise has been shown to influence both inhibitory control and craving. While the evidence for exercise in general is weaker for alcohol than for cocaine dependence, the single study to date to examine the effects of acute bouts on craving found reductions for both substances. Consequently, the aim of this study is to examine whether, following acute exercise bouts at moderate and high intensities, reduced craving and increased inhibitory control can be observed in alcohol or cocaine-dependent individuals. As the focus of this study is the potential of exercise to be integrated into treatment, individuals in treatment for their dependence, who have been abstinent for a short period, will be recruited.

Based on the state of research to date, we propose the following hypotheses:

1. In alcohol-dependent and cocaine-dependent participants, compared to a passive control condition, a bout of moderate exercise will:
  - reduce self-reported craving;
  - improve behavioural performance on the Stroop test;
  - elicit increased activity in the prefrontal cortex during the Stroop test; and
  - be followed by decreased activity in the prefrontal cortex during exposure to substance-related cues.

There is no study which has satisfactorily isolated the effects of intense exercise on craving for alcohol or cocaine. Although Grandjean da Costa et al. [29] found reductions in craving and no increase in inhibitory control during a maximal exercise test, we hold that our protocol, which separates exercise intensities and calls for the Stroop test to be administered only after exercise, may allow for the findings from the literature on exercise and cognition to be identified here, if they occur at all. Consequently:

1. In alcohol-dependent and cocaine-dependent participants, compared to a passive control condition, a bout of intense exercise will:
  - not affect self-reported craving;
  - reduce behavioural performance on the Stroop test;
  - elicit reduced activity in the prefrontal cortex during the Stroop test; and
  - show no differences in activity in the prefrontal cortex during exposure to substance-related cues.

We do not expect that healthy controls will show significantly altered responses to substance cues following any of the experimental conditions.

## Methods

### Ethical approval

Approval for the study (Code 2017–00543) was granted by the intercantonal ethics committee responsible for research at the University of Basel, the Ethikkommission Nordwestschweiz. Any protocol modifications will be communicated to this organisation and the trial sponsor, and registered on ClinicalTrials.gov.

### Study design

This study is a randomised controlled trial involving three conditions: moderate exercise, intense exercise, and rest. Alcohol-dependent, cocaine-dependent and healthy control subjects will undergo all three conditions in randomised order. The order will be determined using a computer-generated ordering system. The random sequence was generated by the web-based Research Randomizer (randomizer.org), which yielded 60 permutations of the order of sessions (rest, moderate exercise, intense exercise). These permutations were printed, placed in separate envelopes and given to a colleague not involved in the study to distribute upon recruitment of each participant. Due to the nature of the intervention, blinding will not take place. The funder has no role in the design, analysis or interpretation of the study.

No data monitoring committee is involved in this study as the intervention (exercise) is not untested or high risk, and as only two acute sessions are involved any adverse events will be addressed immediately by the investigative team. The study therefore meets the criteria of “non-critical indications where patients are treated for a relatively short time and the drugs [in this case, exercise] under investigation are well characterized and known for not harming patients” [41].

### Participants

Twenty alcohol-dependent, 20 cocaine-dependent and 20 healthy control individuals will be recruited to take part in the study (for power calculation, please see “Statistics”). Patients from the inpatient and outpatient branches of the clinic will be informed about the study by physicians, fliers and, in the case of the inpatient clinic, periodic visits from the investigators. All individuals receiving treatment for one of the substance use disorders in question will be approached, in order to ensure that as many individuals as possible who fit the inclusion criteria are reached. The mean age and gender distribution of the cocaine-dependent and alcohol-dependent participants combined will be used as a guide for finding matched healthy controls. Alcohol-dependent and cocaine-dependent individuals will be recruited from the University Psychiatric Clinic of the University of Basel.

Inclusion criteria for these individuals are: age between 18 and 75 years; meeting three or more of the DSM-IV criteria for substance dependence (as determined by the treating physician); minimum of 20 days and maximum of 40 days abstinence from substance of dependence; able to understand and complete the informed consent; able to travel to study site independently, as confirmed by the treating psychiatrist; assessed for physical and mental fitness by the treating physician; and cleared to take part in the study.

The healthy control group will be recruited through personal contacts (the friends and family members of the investigative team and other employees of the Department for Sport, Exercise and Health) and matched for age and gender. Inclusion criteria for the healthy control group are: age between 18 and 75 years; fewer than 3 h of physical exercise or sport per week; and no history of problematic alcohol or illicit drug consumption. All participants will receive a written document outlining the requirements of the study, and informing them that withdrawal at any point will not adversely affect their medical treatment. Participants who agree with the terms will sign the adjacent consent form in the presence of a member of the investigative team, who can also answer any questions arising during the consent process.

Participants will receive a financial incentive of 150 CHF for completion of all three measurements periods, and travel costs will be refunded. Participants will continue to receive treatment as usual during the study phase. In order to optimise participant retention, participants will be contacted by telephone if any of the three scheduled sessions are missed, in order to ascertain, if possible, whether a new session can be scheduled or whether the participant wishes to cease their involvement in the study. Participants may also request telephone or text message reminders of their scheduled sessions.

Prior to the baseline session, all participants will undergo a resting echocardiogram on arrival at the test laboratory. Any participants with questionable findings will be excluded from further participation and referred to their treating physician. Further exclusion criteria include: illness during the study period; inability to complete either exercise condition; and failure to attend three sessions at a maximum of 4 days apart. Participants are not prohibited from any activities during the trial. Any adverse events occurring during the trial will be documented, submitted to the treating physician and ethical committee, and may, upon discussion with the treating physician, lead to exclusion of the participant from the trial.

Data for all participants will be stored only at the intervention site, in coded form, with the key only available to the onsite investigative team. No data from participants who do not complete the full study will be retained or analysed. A single member of the team is responsible for coding, and the code key is locked in a single office at the investigation site. Data entered electronically are not linked in any way to this code list; hard copies of questionnaires are stored in a separate archive. Data entry will be controlled by the principal investigator following entry by other investigators, comparing hard copies with electronic records. Following the trial, archived material will be kept for 10 years, in line with the requirements of the local ethics committee. An inventory of each section of the trial is visible in the SPIRIT checklist (Additional file 1).

### Timeline of assessments

**Fig. 1:**
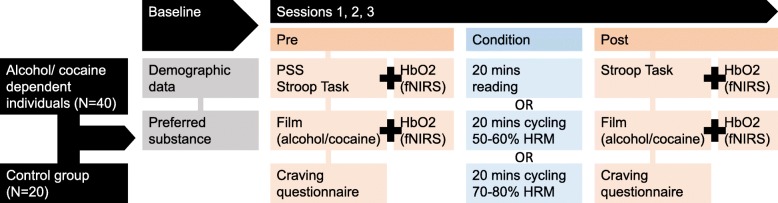
Schedule for each participant in each condition. fNIRS functional near-infrared spectroscopy, HbO_2_ oxygenated haemoglobin, HRM maximal heart rate, PSS Perceived Stress Scale

Figure 1 demonstrates the proposed schedule of assessments for each individual participant.

**Fig. 2:**
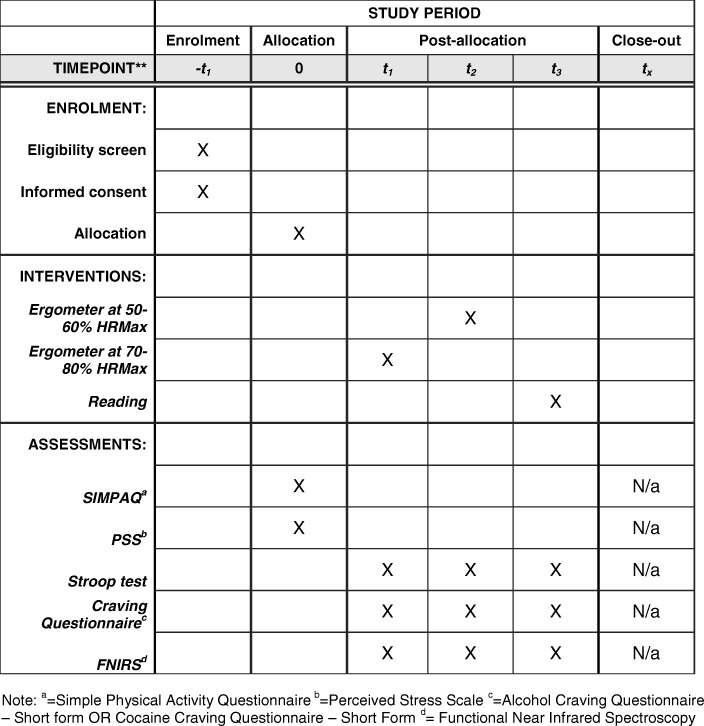
Example of timeline of assessments for a single participant

Figure 2 shows the SPIRIT figure for a single participant in the study.

### Measures

#### Demographic data

The age and gender of all participants will be recorded. Alcohol-dependent and cocaine-dependent participants will be asked to report the year of onset of their dependence, the number of treatment attempts made to date, the number of days of the current treatment episode, the number of days of abstinence and their nicotine consumption.

#### General physical exercise and activity

The SIMPAQ is a brief five-item tool, which comprehensively evaluates activity over the past 7 days including time in bed, sedentary time, time spent walking, type of and time spent in exercise, and time spent for other activities [42]. Assessed physical activity refers to all domains of activity, including leisure time, domestic, work and transport-related activities. The SIMPAQ captures a 24-h period representative for the previous week.

It is important to assess the physical activity levels of participants, as more active individuals may not experience clear changes in prefrontal activation following an acute exercise bout.

### General stress

In order to assess a participant’s stress levels on each test day, a short questionnaire will be completed at the beginning of each session. High stress may increase craving for substances [43, 44], and this questionnaire will allow for this factor to be considered as a covariate. The Perceived Stress Scale will be used to measure the degree to which situations in one’s life are appraised as stressful [45]. It consists of 10 items, each of which can be rated between 0 and 4 on a Likert-type scale, assessing the perceived stress of each participant over the past 4 weeks. Items are summed to generate a single score of between 0 and 40, with higher scores indicating greater perceived stress. The 10-item version of the Perceived Stress Scale is widely reported as having good internal consistency and validity [46].

### Inhibitory control

For the assessment of inhibitory control, a computer-based version of the Stroop test is administered with E-Prime 2.0 (PST, USA). During the task, participants are presented with a colour word appearing in the same colour (e.g. “blue” printed in blue) on compatible trials or in a different colour (e.g. “blue” printed in green) on incompatible trials. While the word meaning has to be ignored, participants are instructed to press a key corresponding to the ink colour. The conflict resulting from the presentation of the relevant and irrelevant dimensions of the stimuli challenges the attentional system. In this respect, a system that efficiently suppresses task-irrelevant dimensions allows fewer conflicts between the colour word and the ink colour. This indicates greater efficiency of the inhibitory control system. Similar to the original Stroop paradigm, computerised versions of this task have been found to elicit interference effects and to have a high reliability [47].

In the present study, participants complete 12 practice trials, followed by four test blocks with 54 trials each. Sufficient practice rounds are included to reduce learning effects. In each test block, only compatible or incompatible trials are presented to allow the detection of interference effects using fNIRS. The blocks are alternated and the trial type employed in the first block is counterbalanced across participants. Between blocks there is a 30-s fixation period, which allows HbO_2_ concentrations to return to baseline. During each trial, the stimuli are presented over 2000 ms or until a response is collected. The inter-stimulus interval is varied randomly between 200 and 400 ms to avoid guessing.

The different colour words were presented with equal probability across all trials. For the assessment of task performance, the accuracy and reaction time (on response-correct trials) are calculated for incompatible trials.

### Alcohol or cocaine cue exposure

Alcohol-dependent participants will be asked to report the type of alcohol (wine, beer, schnapps) which they chiefly consume or prefer. It has been established that, in research involving substance cues, craving is more reliably elicited by cues specific to the individual and their consumption history, and is consequently not a reaction to arousing cues in general [48]. Cocaine-dependent participants will not undergo this step, as there is no variation in the substance itself. All participants will be shown a block of six 1-min films, alternating cocaine cues or alcohol cues (of the preferred substance) with neutral cues. Neutral cues have to be included to be able to associate changes in HbO_2_ with craving rather than visual processing alone. Each 1-min film is separated from the next by a 30-s fixation period, which allows HbO_2_ concentrations to return to baseline. The same film block will be shown at every time point (specifically, prior to and after every condition, for each of the three conditions) in order to ensure that there is no variation in the emotional intensity of the cue.

Each 1-min film is designed to replicate activities carried out in a real-life setting. In order to achieve this, the films are created with a small camera strapped to the head, so that the viewer sees everything from the perspective on the camera holder and can see their hands picking up objects, etc. In the alcohol-cue films, the camera holder walks through a supermarket, arrives at the aisle selling the preferred beverage of the participants, looks at the bottles, picks some up and finally puts some in a basket before moving on. In the neutral films shown following the alcohol films, this sequence is repeated but some type of cleaning product is the examined and chosen. In the cocaine-cue films, the camera holder walks to a flat surface, takes out a small bag of cocaine and begins to snort it or prepares it in lines. The neutral cues are filmed in the same environment, but another activity (making tea, writing, opening a book) is undertaken on the flat surface.

Prior to the study start, the films will be tested on 10 alcohol-dependent and 10 cocaine-dependent individuals (who will not later take part in the study) in order to ensure that the film reliably elicits craving.

### Craving self-report

Current craving levels will be assessed using the Alcohol Craving Questionnaire—Short Form [49] or the Cocaine Craving Questionnaire—Brief [50]. The Alcohol Craving Questionnaire—Short Form consists of 12 questions rated on a 7-point Likert-type scale, addressing the degree to which craving is experienced by the respondent in the current moment. A general craving score is generated by summing the scores of each item (three items are reverse-scored) to produce a score between 12 and 84, with higher scores indicating greater craving. The Short Form has been shown to have good levels of reliability [51] and internal consistency [52]. The Cocaine Craving Questionnaire—Brief consists of 10 items which assess current levels of craving for cocaine. The respondent rates each item on a 7-point Likert-type scale and scores are summed to produce a final value ranging between 7 and 70, with higher scores indicative of greater craving. The questionnaire is reported to have good reliability and internal consistency [53].

### fNIRS recordings

Using fNIRS (NIRSport, NIRx Medizintechnik GmbH, Germany), cerebral oxygenation is recorded during the inhibitory control task and alcohol cue exposure. Probes are applied using a flexible cap adjustable to the participant’s head size. Light sources and detectors are placed over the prefrontal region using the Fz position of the EEG as reference. Channel distances are fixed to 35 mm to allow measurements at equal depths. Offline processing of the collected data is performed with Homer 2 [54] and includes the following steps: removal of discontinuities, artefact rejection and correction using spline interpolation, band-pass filtering (0.01–0.2 Hz), conversion into haemodynamic states using the modified Beer–Lambert Law [55] and normalisation using the pre-task period as baseline. As findings of a review have indicated widespread prefrontal cortex hypoactivation during inhibitory control tasks and hyperactivation during the presentation of drug-related cues, cerebral oxygenation is examined across channels placed over the prefrontal region [7].

HbO_2_ is calculated as the dependent variable, because Strangman et al. [56] have shown high correlations between this parameter and the blood oxygen level. For the assessment of cue-elicited craving, difference waveforms (created by subtracting the haemodynamic response to neutral cues from alcohol/cocaine cues) are extracted for statistical analysis.

### Experimental conditions

To estimate the maximal heart rate of each participant, the algorithm 211 – (0.64 × age) will be used [57].

All participants will undergo three conditions, on three separate days, in a counterbalanced order. The days on which participants participate will be separated by 2–4 days. All sessions will take place in the same laboratory room at the Department of Sport, Exercise and Health at the University of Basel. Trained members of the investigative team will oversee all conditions. Owing to the nature of the intervention, investigators will not be blinded to the conditions.

The reading condition serves as a resting state comparison. Subjects will be asked to read an article about architecture in Basel. The article is 13 pages long, excluding references.

The moderate exercise condition requires participants to train on a cycle ergometer at between 50 and 60% of their maximal heart rate. Participants will wear a heart rate monitor, which will be constantly observed by study personnel, to ensure that they exercise with the correct intensity. If the participant exceeds or fails to meet the appropriate intensity, prompts will be given. Prompts will not be recorded. This constitutes a moderate, aerobic exercise bout. The exercise will last 20 min.

The intensive exercise condition is identical to the moderate condition, but participants will exercise at a pulse of between 70 and 80% of their maximal heart rate, constituting a bout approximately at the anaerobic threshold.

### Statistics

Sample size is calculated a priori using G*Power 3.1 [58]. Based on effect sizes reported in previous studies [25, 40] and an alpha level set to 0.05, the initial power analysis indicates that 17 participants per group are required to reach 85% statistical power. As drop-outs have to be expected, the sample size is increased to 20 participants per group. Note that the power analysis is based on the acute effects of moderate aerobic exercise on prefrontal cortex activity and inhibitory control. The statistical analysis of collected data is performed with SPSS 23.0 (IBM Statistics, USA) for Windows. Acute effects of exercise on inhibitory control and HbO_2_ concentration are examined using a 2 (group: alcohol dependent, healthy controls) × 3 (condition: control, moderate exercise, highly intense exercise) × 2 (time: pre, post) ANOVA on reaction time and the haemodynamic response to incompatible trials of the Stroop colour–word test. Additionally, effects of exercise on cue-elicited craving and HbO_2_ concentration (neutral cues subtracted from alcohol/cocaine cues) are investigated by applying a 2 (group) × 3 (condition) × 2 (time) ANOVA. Main effects and interactions are reported. Subsequently, Bonferroni-corrected *t* tests are used to decompose any significant interactions. The level of statistical significance is set to *p* < 0.05. Data from participants who do not complete all three measurement points will not be used.

Regarding missing data, as participants who do not complete all three sessions will be excluded from the study, it is anticipated that missing data will chiefly be due to faults in the fNIRS and Stroop programming, and thus will be missing at random. For this reason, multiple imputation will be used to address this issue.

### Dissemination of results

Only the investigative team will have access to the trial dataset. Results of the trial will be communicated to participants following completion of the study upon request. Individual results will not be made available. Physicians from the clinics involved in recruiting the patients will be informed about the results, and upon request the investigators will present these at faculty meetings. Findings from the study will be published in peer-reviewed academic journals.

## Discussion

To date, it has been shown that a maximal exercise test leads to reduced alcohol or cocaine craving, but not improvements in inhibitory control, when the inhibitory control test is taken during exercise [29]. Using fNIRS is a potentially promising method of imaging neural activity during both craving and inhibitory control tasks, and may provide more information about the mechanisms of exercise than craving self-report and test performance alone [23]. Furthermore, if found to be a reliable method, fNIRS can be much more easily employed in clinical practice than fMRI and EEG.

Positive effects of acute exercise bouts on alcohol craving have been reported elsewhere. Ussher et al. [59] found that 10 min of moderate cycle ergometry led to reduced craving compared with non-exercising controls, although, interestingly, differences disappeared following exercise. A finding of reduced craving following either exercise condition in the present study might therefore be an initial indicator of the duration required to elicit effects. There is no second study, to the best of our knowledge, addressing exercise and cocaine craving; a pre-clinical study assessing cocaine-seeking behaviour in rats found that 2 h of wheel-running, compared to no exercise, led to significant reductions [60].

The entirety of the literature on exercise (both acute and long-term interventions) as a treatment for alcohol dependence [34, 61] is somewhat less positive than that for illicit drugs [33, 62] and nicotine [63], although it must be noted that high-quality studies are still lacking for all substances. However, regarding cocaine specifically, the sole study to differentiate this substance from an umbrella term of “various drugs” is that of Grandjean da Costa et al. [29]. The findings of the planned study may be a further contribution towards developing studies and programmes aimed at targeting specific substance use disorders, one of the next steps in the research on exercise in this field. It will also be important to address the question of why exercise for alcohol dependence has so far yielded mixed results; is a reduction in craving indeed so short-lived that it stops immediately after exercise, or is even a longer-lasting reduction not sufficient to impact on changes in consumption?

A key aspect of the current study is the question of whether inhibitory control moderates the effects of exercise on craving. Despite research indicating that exercise at moderate intensities improves performance on inhibitory control tasks, such as the Stroop test, Grandjean da Costa et al. [29] reported no significant improvements. A non-significant trend towards improvement was reported; and, as already noted, participants in that study performed the Stroop test while exercising. The pattern of results shows that, descriptively, participants’ best performances were achieved at baseline, although one would expect improvements over repeated measurements, especially while exercising at a moderate intensity [64, 65]. It may be that, when participants are not trying to complete both exercise and the test, results at moderate intensities improve; however, this study did report reductions in craving, so until different permutations of the exercise/inhibitory control test combinations are tested, any commentary is merely speculation. We believe that in spite of this finding, the literature on both exercise improving inhibitory control and inhibitory control being associated with substance use disorders is robust, and therefore warrants exploration with new study protocols.

Already noted, and decisive in the formulation of our hypotheses, is the fact that no study to date has compared separate exercise bouts at clearly defined intensities with the goal of eliciting their effects on craving for alcohol or cocaine. Consequently, it cannot be determined whether any type of exercise is enough to affect craving, or whether reductions are the result of particular intensities. Such an investigation has been carried out for methamphetamine, with the result that both moderate and intense exercise bouts reduced craving equally during and 50 min after exercise, and to a significantly greater degree than light or no exercise [66]. However, immediately after exercise, craving was lower following the intense bout than the moderate bout. The test of inhibitory control, completed 20 min after exercise, showed the fastest reaction times following the moderate bout. An assessment of inhibitory control immediately after exercise in this study might have shed more light on the role of inhibitory control in craving; we have therefore designed our study so that craving self-report and fNIRS assessment always take place immediately prior to the Stroop test.

A final key issue that this study aims to shed further light on is the value of fNIRS assessments in addiction treatment. In the past 2 years, this technique has been used in the assessment of anxiety in heroin users [67], working memory in ecstasy users [68] and investigations of gambling [69] and Internet addiction [70]. While the simplicity, cost-effectiveness and lack of physical restrictiveness noted earlier are all attractive features of the device, it must be recognised that the bulk of the limbic system, which plays a crucial role in substance addiction [71], cannot be imaged using fNIRS. In order to maintain reasonable signal-to-noise ratios, only cortical brain activity (2–6 mm into cerebral tissue) can be mapped using fNIRS. Furthermore, recent research on the fNIRS methodology suggested that systemic changes in the extracerebral and intracerebral blood flow might compromise the interpretation of measured fNIRS signals (which is also true for other haemodynamic-based techniques such as fMRI) [72]. However, with a thorough experimental design and precise instructions to the study participant, these potential confounding factors can be kept to a minimum. Through its advantages especially in applications that require high ecological validity, and due to its high level of consistency with traditional neuroimaging techniques, fNIRS has a great potential to expand our knowledge on the functional organisation of the brain [73].

### Trial status

Ongoing. Patient recruitment incomplete.

## Additional file

Additional file 1: SPIRIT 2013 checklist: recommended items to address in a clinical trial protocol and related documents (DOC 124 kb)
